# Supplementary material for: Evidence supported by Mendelian randomization: impact on inflammatory factors in knee osteoarthritis
Source: Front Med (Lausanne). 2024 May 28;11:1382836. doi: 10.3389/fmed.2024.1382836 (PMC11165061; doi:10.3389/fmed.2024.1382836)
Supplement: Supplementary file 1 [file Data_Sheet_1.ZIP › data/GCST90274841_5e6/841.leaveoneout.pdf]

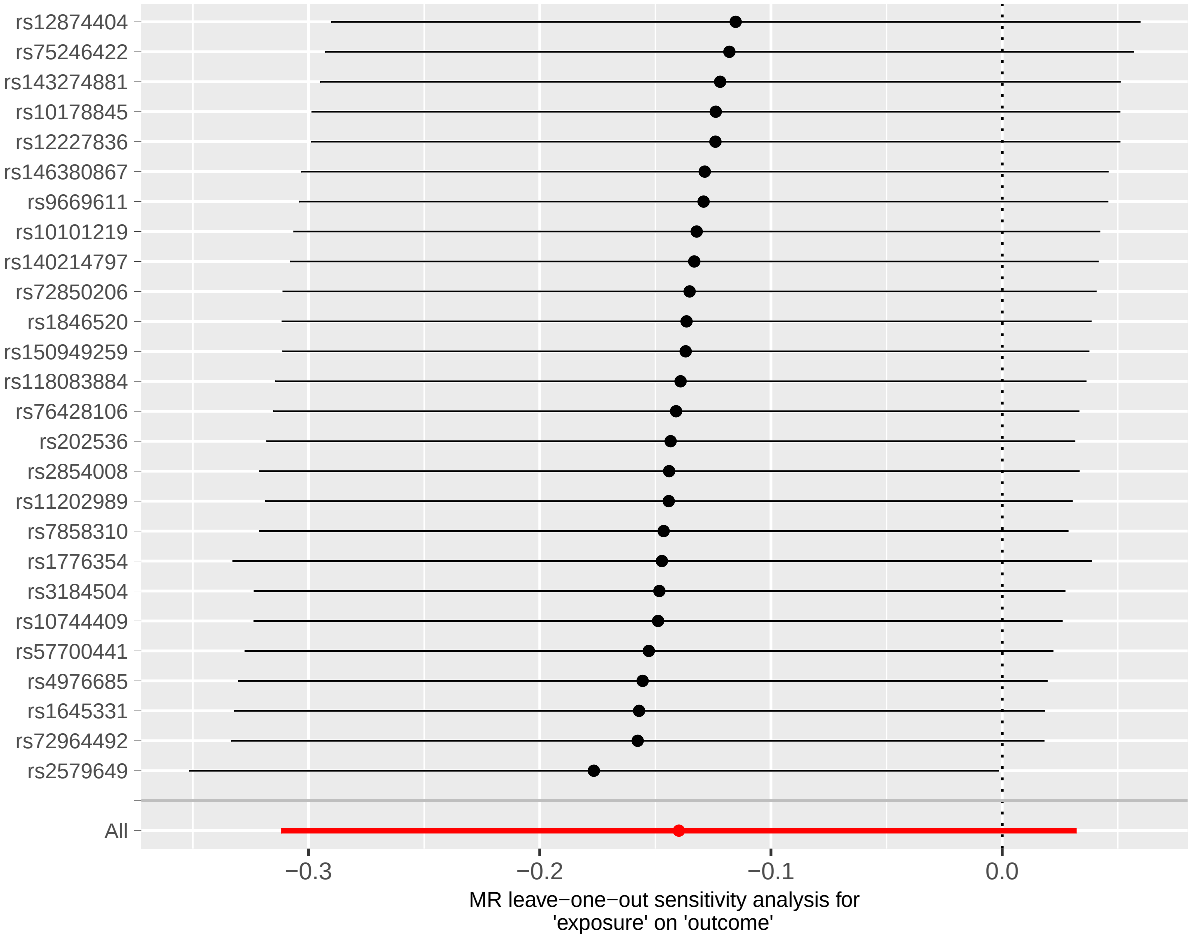
rs12874404
rs75246422
rs143274881
rs10178845
rs12227836
rs146380867
rs9669611
rs10101219
rs140214797
rs72850206
rs1846520
rs150949259
rs118083884
rs76428106
rs202536
rs2854008
rs11202989
rs7858310
rs1776354
rs3184504
rs10744409
rs57700441
rs4976685
rs1645331
rs72964492
rs2579649
All
−0.3
−0.2
−0.1
0.0
MR leave−one−out sensitivity analysis for
'exposure' on 'outcome'
